# Short-term outcomes of robotic-assisted versus laparoscopic inguinal hernia repair: a single-center retrospective study in Taiwan

**DOI:** 10.3389/fsurg.2026.1763011

**Published:** 2026-08-25

**Authors:** Ching-Lung Hsieh, Fang-Rong Hsu, Cheng-Ming Peng, Teng-Chieh Cheng

**Affiliations:** 1 Department of Information Engineering and Information Science, Feng Chia University, Taichung, Taiwan; 2 Department of General Surgery, Da Vinci Minimally Invasive Surgery Center, Chung Shan Medical University Hospital, Taichung, Taiwan; 3 College of Medicine, Chung Shan Medical University, Taichung, Taiwan

**Keywords:** inguinal hernia repair, laparoscopic, outcomes, robotic, surgery

## Abstract

**Introduction:**

Robotic-assisted surgery has emerged as an alternative to conventional laparoscopy for inguinal hernia repair; however, comparative evidence from Asian populations remains limited. This study compared the short-term outcomes of robotic-assisted and laparoscopic inguinal hernia repair at a tertiary medical center in Taiwan.

**Methods:**

This single-center retrospective observational study included adults who underwent elective robotic-assisted or laparoscopic inguinal hernia repair between June 2020 and January 2023. Demographic characteristics, comorbidities, hernia characteristics, operative details, and postoperative outcomes were extracted from electronic medical records. The primary outcome was postoperative complications. Secondary outcomes included hernia recurrence, reoperation, 30-day readmission, operative time, intraoperative blood loss, postoperative analgesic use, and length of hospital stay. Between-group comparisons were performed using Student's t-test, the Wilcoxon rank-sum test, the Chi-squared test, or Fisher's exact test, as appropriate.

**Results:**

Of 105 patients screened, 58 met the inclusion criteria; 13 underwent robotic-assisted repair and 45 underwent laparoscopic repair. Robotic-assisted procedures were more frequently performed using the transabdominal preperitoneal approach, whereas laparoscopic procedures predominantly used the totally extraperitoneal approach (69.2% vs. 6.7% for transabdominal preperitoneal repair, respectively; *p* < 0.001). Suture fixation was more common in the robotic group, whereas absorbable tack fixation predominated in the laparoscopic group (*p* < 0.001). Mean operative time was similar between the robotic and laparoscopic groups (146.8 ± 42.9 vs. 145.0 ± 39.9 minutes; *p* = 0.890). No conversions, transfusions, or intraoperative complications occurred. No significant differences were observed in postoperative complications, recurrence, reoperation, 30-day readmission, length of hospital stay, or duration of intravenous analgesic use.

**Discussion:**

Robotic-assisted inguinal hernia repair was associated with similar observed short-term outcomes to conventional laparoscopic repair. However, the small robotic cohort, retrospective design, group imbalance, and limited follow-up reduced the ability to detect differences in uncommon or long-term outcomes. Larger prospective studies with longer follow-up are required to clarify the potential benefits of robotic-assisted repair, particularly in complex or recurrent cases.

## Introduction

Inguinal hernia is among the most common conditions encountered in general surgical practice, comprising 7% of all surgical outpatient visits (1). Worldwide, over 20 million inguinal hernia repairs are performed annually, underscoring its significance as one of the most frequently conducted surgical procedures (2). It is estimated that approximately 27% of men and 3% of women will develop an inguinal hernia during their lifetime (3). In Taiwan, inguinal hernia repair accounts for a considerable proportion of elective general surgery cases. Over the past 2 decades, there has been a marked shift toward minimally invasive surgical approaches, largely driven by the pursuit of improved patient outcomes and recovery experiences.

Traditional open hernia repair has gradually been complemented by laparoscopic techniques, particularly transabdominal preperitoneal (TAPP) and totally extraperitoneal (TEP) repairs (2). These minimally invasive methods have demonstrated comparable safety and efficacy to open repair, with added benefits including reduced postoperative pain, shorter hospital stays, faster return to normal activity, and better cosmetic results (4). As laparoscopic repair continues to gain prominence, a new frontier has emerged in the form of robotic-assisted surgery. In recent years, robotic-assisted hernia repair has been increasingly adopted, particularly in centers with established robotic platforms and growing experience in minimally invasive abdominal wall surgery (5). Its utilization has expanded alongside broader dissemination of robotic systems in general surgery, although the degree of adoption varies substantially across regions and healthcare systems.

Robotic-assisted inguinal hernia repair builds upon the principles of laparoscopy, incorporating advanced technological features such as high-definition 3-dimensional (3D) visualization, enhanced dexterity through articulated instruments, and tremor filtration (6). Nonetheless, the integration of robotic systems into routine hernia repair remains controversial. While some studies suggest comparable or even superior short-term outcomes relative to conventional laparoscopy (7, 8), others highlight potential drawbacks such as increased operative times, steep learning curves, and higher associated costs (9, 10). Recent systematic reviews and meta-analyses have similarly reported that robotic and laparoscopic inguinal hernia repairs generally provide comparable short-term postoperative outcomes, including complications, postoperative pain, conversion, and recurrence (11); however, robotic repair is often associated with longer operative time, particularly for unilateral hernias, and higher procedural costs. These findings suggest that the incremental benefit of robotic repair over conventional laparoscopy remains uncertain, especially from the perspectives of operative efficiency and cost-effectiveness. Moreover, much of the existing evidence originates from Western populations, limiting its generalizability to Asian healthcare settings, where variations in patient body habitus, surgical training, and perioperative management may influence outcomes.

In Taiwan, data comparing robotic-assisted and laparoscopic inguinal hernia repair is limited. Specifically, there is a lack of studies evaluating detailed intraoperative variables, mesh fixation techniques, and short-term postoperative outcomes within the local context. Addressing this gap, the present study aims to compare the short-term outcomes of robotic-assisted vs. laparoscopic inguinal hernia repairs performed at a single tertiary medical center in Taiwan. By examining patient characteristics, operative details, and early postoperative recovery, this retrospective analysis seeks to provide locally relevant evidence to inform surgical decision-making, optimize resource utilization, and support the evolving role of robotic surgery in the management of inguinal hernias.

## Methods

### Study design and setting

This was a single-center, retrospective observational study conducted at the Department of General Surgery, Chung Shan Medical University Hospital, Taichung, Taiwan. The study was approved by the Institutional Review Board of Chung Shan Medical University Hospital. The requirement for informed consent was waived due to the retrospective nature of the study and the use of de-identified data.

### Ethics statement

The study protocol was approved by the Institutional Review Board (IRB) of Chung Shan Medical University Hospital (IRB No. CS1-22195). Given the retrospective design and use of de-identified data, the IRB waived the requirement for informed consent.

### Study population

All adult patients aged 18 years old or older who underwent laparoscopic or robotic-assisted inguinal hernia repair between June 2020 and January 2023 at a tertiary center, Chung Shan Medical University Hospital, in Taiwan were screened for eligibility.

All included patients were elective cases managed in the outpatient setting. Patients were eligible for inclusion if they had a diagnosis of unilateral (left or right) or bilateral inguinal hernia. Patients with incarcerated hernia were considered eligible for minimally invasive repair only when the hernia contents were reducible, particularly in cases of omental incarceration. Patients with irreducible bowel-containing hernia, bowel obstruction, or suspected strangulation were not considered candidates for laparoscopic or robotic-assisted repair and were managed with open surgery; therefore, such cases were not included in this cohort.

Patients were excluded if they did not have an inguinal hernia or if the inguinal hernia was combined with other types of hernia, such as incisional, hiatal, umbilical, femoral, or obturator hernia.

### Data collection

Clinical data were extracted from electronic medical records and included demographic characteristics (age, sex, height, weight), comorbidities [American Society of Anesthesiologists (ASA) physical status classification and Charlson Comorbidity Index (CCI)], hernia type and size, history of lower abdominal surgery, and operative variables such as surgical approach, mesh type, mesh fixation method, operative time, console time (for robotic procedures), intraoperative blood loss, and intraoperative complications.

### Surgical techniques

All procedures were performed by two experienced surgeons. Patients underwent either laparoscopic TAPP or TEP, or robotic-assisted TAPP or TEP inguinal hernia repair, depending on surgeon preference, patient factors, and logistical considerations. In general, the choice between robotic and laparoscopic repair was made after discussion with the patient regarding the expected risks, benefits, and cost differences between approaches. Robotic repair was more commonly selected for patients who preferred this approach and agreed to the higher out-of-pocket expenses. From a logistical perspective, robotic surgery also depended on operating room scheduling and the availability of a dedicated robotic surgical team, including trained nursing staff; therefore, robotic procedures were typically performed during regular weekday operating hours. Cases presenting during weekends or requiring more urgent intervention were more likely to be managed using laparoscopic or open approaches. Mesh selection, fixation method, and the use of anti-adhesion agents were determined intraoperatively by the attending surgeon. All robotic procedures were performed using the da Vinci Xi surgical system.

### Outcome measures

The primary outcome measure was the occurrence of postoperative complications during the follow-up period. Secondary outcome measures included hernia recurrence, reoperation, and readmission within 30 days, as well as operative time, console time (robotic group), intraoperative blood loss, postoperative analgesic use (type, duration, and opioid requirement), and length of hospital stays. Postoperative follow-up was conducted through routine outpatient clinic visits, generally at approximately 3-month intervals. Hernia recurrence was assessed based on patient-reported symptoms and physical examination findings. Imaging studies were arranged when recurrence or mesh-related complications were clinically suspected. Patients were not systematically contacted outside routine clinical visits.

### Statistical analysis

Continuous variables with a normal distribution were presented as mean ± standard deviation (SD) and were analyzed using Student's t-test; continuous data without normal distribution were presented as median (interquartile range [IQR]) and were analyzed using the Wilcoxon rank-sum test. Categorical variables were expressed as counts and percentages and analyzed using the Chi-squared test or Fisher's exact test, as appropriate. All statistical analyses were performed using SAS version 9.4 software (SAS Institute Inc., Cary, NC, USA). A 2-sided *p*-value of < 0.05 was considered statistically significant.

## Results

### Study population selection

A total of 105 patients who underwent hernia repair were initially enrolled. Among them, 36 patients were excluded due to having a hernia other than an inguinal hernia (e.g., umbilical, femoral, hiatal, diaphragmatic, or incisional hernias), or having an inguinal hernia combined with other types of hernia, and 11 patients with recurrent (preoperative) hernia were also excluded. Finally, 58 patients with unilateral or bilateral inguinal hernia were included in the analysis. Of these, 13 patients underwent robotic surgery, and 45 patients underwent laparoscopic surgery.

### Patient characteristics

The patient characteristics, and surgical and operative information are summarized in Tables 1, 2. The overall mean age was 57 years, and 82.8% were male (Table 1).

**Table 1:** Patient characteristics.

Characteristics	Total	Robotic surgery	Laparoscopy	*p*-value
	(*N* = 58)	(*n* = 13)	(*n* = 45)	
Demography				
Age, years	57.0 ± 16.7	61.8 ± 17.2	55.6 ± 16.5	0.247
Sex				0.097
Male	48 (82.8)	13 (100.0)	35 (77.8)	
Female	10 (17.2)	0 (0.0)	10 (22.2)	
BMI, kg/m^2^	23.9 ± 3.7	23.1 ± 2.5	24.1 ± 3.9	0.367
ASA				1.000
I	12 (20.7)	3 (23.1)	9 (20.0)	
II	41 (70.7)	9 (69.2)	32 (71.1)	
III	5 (8.6)	1 (7.7)	4 (8.9)	
Pre-operative hernia size (optional variable)				0.915
<1.5 cm (1 finger)	14 (24.1)	3 (23.1)	11 (24.4)	
<3 cm (2 fingers)	30 (51.7)	6 (46.2)	24 (53.3)	
>3 cm (>2 fingers)	14 (24.1)	4 (30.8)	10 (22.2)	
History of lower abdominal surgery	5 (8.6)	0 (0.0)	5 (11.1)	0.577
Charlson Comorbidity Index				0.225
0	34 (58.6)	5 (38.5)	29 (64.4)	
1	14 (24.1)	5 (38.5)	9 (20.0)	
2+	10 (17.2)	3 (23.1)	7 (15.6)	

Continuous data are presented as mean ± standard deviation or median (25th −75th percentile). Categorical data are presented as count (percentage).

**Table 2:** Surgical and intraoperative data.

	Total	Robotic surgery	Laparoscopy	*p*-value
	(*N* = 58)	(*n* = 13)	(*n* = 45)	
Surgical information				
Surgical technique				**<0**.**001**
TAPP	12 (20.7)	9 (69.2)	3 (6.7)	
TEP	46 (79.3)	4 (30.8)	42 (93.3)	
Laterality				0.515
Bilateral	37 (63.8)	7 (53.8)	30 (66.7)	
Unilateral	21 (36.2)	6 (46.2)	15 (33.3)	
Inguinal hernia type				0.102
Direct	9 (15.5)	4 (30.8)	5 (11.1)	
Indirect	49 (84.5)	9 (69.2)	40 (88.9)	
Mesh fixation				**<0**.**001**
Absorbable tack	49 (84.5)	5 (38.5)	44 (97.8)	
Glue	1 (1.7)	0 (0.0)	1 (2.2)	
Suture	6 (10.3)	6 (46.2)	0 (0.0)	
No fixation	2 (3.4)	2 (15.4)	0 (0.0)	
Hernia type				0.524
Bilateral inguinal hernia	38 (65.5)	7 (53.8)	31 (68.9)	
Left inguinal hernia	9 (15.5)	3 (23.1)	6 (13.3)	
Right inguinal hernia	11 (19.0)	3 (23.1)	8 (17.8)	
Cord lipoma	5 (8.6)	2 (15.4)	3 (6.7)	0.311
Posterior wall weakness	23 (39.7)	8 (61.5)	15 (33.3)	0.067
Hernia defect, cm				0.138
1 × 1	52 (89.7)	10 (76.9)	42 (93.3)	
1 × 2	2 (3.4)	1 (7.7)	1 (2.2)	
2 × 2	3 (5.2)	1 (7.7)	2 (4.4)	
4 × 2	1 (1.7)	1 (7.7)	0 (0.0)	
Mesh size, cm				1.000
10 × 12	1 (1.7)	0 (0.0)	1 (2.2)	
10 × 15	56 (96.6)	13 (100.0)	43 (95.6)	
13 × 15	1 (1.7)	0 (0.0)	1 (2.2)	
Mesh^a^				0.342
Heavyweight	14 (24.1)	1 (7.7)	13 (28.9)	
Lightweight	43 (74.1)	12 (92.3)	31 (68.9)	
Both	1 (1.7)	0 (0.0)	1 (2.2)	
Trocar, piece				0.123
3	55 (94.8)	11 (84.6)	44 (97.8)	
4	3 (5.2)	2 (15.4)	1 (2.2)	
Suture material				**<0**.**001**
Stratafix	11 (19.0)	8 (61.5)	3 (6.7)	
Vicryl	3 (5.2)	2 (15.4)	1 (2.2)	
Not specified	44 (75.9)	3 (23.1)	41 (91.1)	
Anti-adhesion agent (barre gel)	1 (1.7)	1 (7.7)	0 (0.0)	0.224
Intraoperative information				
Operative time, min	145.4 ± 40.2	146.8 ± 42.9	145.0 ± 39.9	0.890
Console time, min	128.9 ± 38.0	125.8 ± 40.2	129.9 ± 37.8	0.735
Blood loss				0.634
5 ml	34 (58.6)	9 (69.2)	25 (55.6)	
10 ml	23 (39.7)	4 (30.8)	19 (42.2)	
105 ml	1 (1.7)	0 (0.0)	1 (2.2)	
Surgery port (Non-single port)	58 (100.0)	13 (100.0)	45 (100.0)	-

TAPP, transabdominal preperitoneal; TEP, totally extraperitoneal.

Continuous data are presented as mean ± standard deviation or median (25th–75th percentile). Categorical data are presented as count (percentage).

A *p*-value < 0.05 is considered statistically significant and is shown in bold.

a Heavyweight refers to B-braun mesh; lightweight includes Covidien mesh and Hernia mesh. The “both” indicates the use of both Covidien mesh and B-braun mesh.

The surgical techniques used differed significantly between the 2 groups: 69.2% of patients in the robotic group underwent TAPP repair, while 93.3% of patients in the laparoscopic group underwent TEP repair (*p* < 0.001). Mesh fixation methods also differed significantly between groups. Suture fixation was used in 46.2% of patients in the robotic group, whereas absorbable tacks were used in 97.8% of patients in the laparoscopic group (*p* < 0.001). A higher proportion of patients in the robotic group received Stratafix and Vicryl suture materials compared with those in the laparoscopic group (*p* < 0.001) (Table 2).

No occurrences of conversion, transfusion, or intraoperative complications (bowel serosal injury, vessel injury, bladder injury, intestinal injury, bowel perforation, and vas deferens injury) were observed during any of the operations.

### Post-operative outcomes

Post-operative outcomes are summarized in Table 3. There were no significant differences in post-operative outcomes between groups. The overall median length of hospital stay was 3 days. A total of 6 patients experienced post-operative complications. In the robotic surgery group, 1 patient had a surgical site infection, and 1 patient had urinary retention/anuria. In the laparoscopy group, 1 patient experienced other rare complication (right scrotum swelling and later diagnosed with angiofibroma), and 3 patients had a hernia recurrence after surgery. Additionally, 2 patients in the laparoscopy group required reoperation; however, these procedures were not due to postoperative complications of the index repair. One patient developed a contralateral inguinal hernia during follow-up and underwent repair as a metachronous event. No cases of postoperative bleeding, hematoma/seroma, postoperative pain with intervention, adhesion, obstruction, fistula, perforation, thigh numbness, 30-day readmission, or mortality occurred in either group. All postoperative complications were minor and corresponded to Clavien–Dindo Grade I–II.

**Table 3:** Post-operative outcomes .

	Total	Robotic surgery	Laparoscopy	*p*-value
	(*N* = 58)	(*n* = 13)	(*n* = 45)	
Post-operative outcomes				
Length of hospital stay, days	3.0 (2.0–3.0)	3.0 (2.0–3.0)	3.0 (2.0–3.0)	0.779
Follow-up time, months	1.0 (0.5–2.0)	1.0 (0.5–2.0)	1.0 (0.5–2.0)	0.930
Post-operative complication	6 (10.3)	2 (15.4)	4 (8.9)	0.608
Surgical site infection	1 (1.7)	1 (7.7)	0 (0.0)	0.224
Rare complication^a^	1 (1.7)	0 (0.0)	1 (2.2)	1.000
Urinary retention/anuria	1 (1.7)	1 (7.7)	0 (0.0)	0.224
Recurrence	3 (5.2)	0 (0.0)	3 (6.7)	1.000
Reoperation	2 (3.4)	0 (0.0)	2 (4.4)	1.000
Medication usage				
Use of IV analgesics	58 (100.0)	13 (100.0)	45 (100.0)	-
Use of IV opioids	56 (96.6)	13 (100.0)	43 (95.6)	1.000
Total days of IV analgesic use	2.0 (2.0–4.0)	3.0 (2.0–4.0)	2.0 (2.0–4.0)	0.336

IV, intravenous therapy.

Continuous data are presented as mean ± standard deviation or median (25th–75th percentile). Categorical variables are presented as count (percentage).

a One patient developed right scrotal swelling and was later diagnosed with angiofibroma.

All patients received intravenous (IV) analgesics postoperatively. The overall median duration of IV analgesic use was 2 days, with no statistically significant difference between the robotic and laparoscopic groups (Table 3).

## Discussion

In this study, robotic-assisted repairs were more frequently performed using the TAPP approach, whereas TEP was predominant in the laparoscopic group. Mesh fixation methods also differed, with suture fixation more commonly used in the robotic group and absorbable tack fixation more common in the laparoscopic group. No conversions, transfusions, or intraoperative complications occurred in either group. Overall, no significant differences were observed in postoperative complications, recurrence, reoperation, 30-day readmission, length of hospital stay, or duration of postoperative intravenous analgesic use. However, given the small number of robotic cases and the short follow-up duration, these findings should be interpreted cautiously.

Numerous studies across different healthcare settings have examined minimally invasive approaches to inguinal hernia repair. This is in part due to the relatively high occurrence in men. Studies have overall reported the various minimally invasive methods are reliable with a low frequency of complications. In addition to prior Taiwanese multicenter and nationwide studies that primarily examined broad outcomes such as reoperation, opioid use, hospital stay, and costs, the present single-center analysis focused on short-term outcomes in routine clinical practice. This provides complementary real-world evidence, with the findings reflecting certain surgical technique preferences, namely, the predominance of TAPP with suture fixation in robotic-assisted repair vs. TEP with tack fixation in laparoscopic repair. Chao et al. (12) reported similar outcomes of robotic and laparoscopic inguinal hernia repair; however, robotic surgery was associated with a lower reoperation rate and less opioid use in complicated cases. Pai et al. (13) reported similar results; however, robotic surgery was associated with a lower complication risk and shorter LOS in obese patients, but the hospital costs were higher.

In a prior study that included 704 patients, Okamoto et al. (14) reported that robotic transabdominal preperitoneal repair (R-TAPP) showed outcomes comparable to those of laparoscopic transabdominal preperitoneal repair (L-TAPP). Similarly, another study showed that R-TAPP achieves similar outcomes as laparoscopic totally extraperitoneal repair (L-TEP), which has the advantage of not entering the abdominal cavity but is more technically demanding than other methods (15). Notably, however, R-TAPP was associated with a significantly decreased hernia recurrence rate. These results are consistent with our findings, which showed comparable observed short-term outcomes between robotic-assisted and laparoscopic repair with respect to complications, recurrence, and postoperative recovery, while highlighting the need for longer-term evaluation. However, the lack of a significant difference in our study may be attributable to the predominance of standard-risk cases in our cohort. Robotic-assisted repair may provide distinct advantages in more complex scenarios, such as recurrent hernias, large hernias, or cases with dense intra-abdominal adhesions (16, 17). In addition, surgeon selection bias and varying learning curves between robotic and laparoscopic techniques may have contributed to the observed outcomes.

In our study, robotic-assisted repairs were more frequently performed using the TAPP approach, whereas laparoscopic repairs predominantly employed TEP. This reflects current practice trends, as a 2024 review indicated that robotic TAPP repairs are performed more frequently, largely because the robotic platform facilitates intracorporeal suturing and navigation within the peritoneal cavity, making TAPP the preferred route in robotic hernia surgery (4). Many studies have reported comparable recurrence and complication rates between laparoscopic TAPP, laparoscopic TEP, and robotic TAPP, with TAPP occasionally associated with slightly higher rates complications that could be managed conservatively presumably due to larger defects and older patient populations (18–20). A meta-analysis comparing TAPP and TEP found that TEP had a higher seroma rate than TAPP, while it has associated with lower scrotal/cord edema rates immediately and at 1 week postoperatively (19). A study with 1-year follow-up showed that the hernia recurrence rates were low at 1 year (≤2.8%) and similar between unilateral and bilateral R-TAPP and between unilateral and bilateral TEP (20).

Our results showed that suture fixation was significantly more common in the robotic group, while laparoscopic repairs predominantly used absorbable tacks, reflecting platform-related technical preferences. Prior studies similarly report that the fixation method mainly affects postoperative pain and cost rather than recurrence, with suture fixation often associated with less long-term pain compared with tacks. Kumar et al. (21) compared mesh suture fixation to tack fixation in patients undergoing TEP. The only difference in postoperative outcomes were lower VAS pain scores at 1 and 3 months in the suture group. A similar study examining suture vs. tack mesh fixation in patients undergoing TAPP reported similar postoperative outcomes, except for higher pain scores in the tack fixation group (22).

In the present cohort, both robotic-assisted and laparoscopic inguinal hernia repairs were associated with low rates of perioperative adverse events and comparable observed short-term postoperative outcomes, as reflected by the absence of conversion, transfusion, and intraoperative complications, as well as no significant differences in postoperative complications, hernia recurrence, reoperation, or 30-day readmission. A meta-analysis similarly concluded that robotic and laparoscopic repairs have similar clinical effects and safety profiles, despite longer operative time in robotic cases (23). Similar to other studies, our results showed that both methods were associated with overall low complication rates and no serious adverse events were observed in either group. Also notable, recovery, as indicated by similar duration of postoperative IV analgesia, was similar between the groups. These observations align with broader evidence suggesting that robotic-assisted and laparoscopic approaches are associated with similar observed safety and recovery profiles in inguinal hernia repair (24).

Operative time is a key consideration when evaluating robotic surgery, particularly given concerns that longer operative duration may increase resource utilization and overall costs. In our study, intraoperative time was comparable between the robotic and laparoscopic groups. This finding suggests that robotic-assisted repair may be performed with similar operative efficiency in our setting, although this should be interpreted cautiously given the small sample size and single-center design.

### Clinical implications

In this cohort, both laparoscopic and robotic approaches were associated with low complication rates and early postoperative recovery. It should be noted that this study excluded complex or recurrent hernias, such as hernias other than inguinal (e.g., umbilical, femoral, hiatal, diaphragmatic, or incisional hernias) and cases with concomitant inguinal and other types of hernias, where the robotic platform may theoretically provide greater technical advantages owing to its enhanced dexterity and 3D visualization, particularly in confined or adhesive operative fields. Further studies focusing on these challenging scenarios are warranted to determine whether robotic repair offers additional clinical benefits beyond conventional laparoscopy.

Looking ahead, the integration of artificial intelligence (AI) with robotic surgical platforms represents a promising direction in minimally invasive surgery. Emerging applications include real-time intraoperative guidance, automated image recognition, workflow optimization, and decision support systems, which may enhance surgical precision and standardization (25). As these technologies continue to evolve, AI-assisted robotic surgery has the potential to further improve clinical outcomes and expand the applicability of minimally invasive approaches in hernia repair and beyond.

### Strengths and limitations

A major strength of this study is that it provides locally relevant evidence from Taiwan, addressing the gap in comparative data between robotic-assisted and laparoscopic inguinal hernia repair in Asian populations. Detailed operative variables, including surgical approach, mesh fixation techniques, and short-term postoperative outcomes were systematically analyzed, offering a comprehensive assessment of perioperative care. Additionally, the study used data from a single tertiary medical center where procedures were performed by experienced surgeons, reducing heterogeneity in surgical technique and perioperative management. However, several limitations should be noted. The retrospective design introduces potential selection bias and limits the generalizability of the findings. The relatively small sample size, particularly in the robotic group, as well as the marked imbalance in group size between the robotic and laparoscopic cohorts, may have reduced the statistical power to detect meaningful differences in relatively infrequent outcomes, such as hernia recurrence and postoperative complications. Therefore, the absence of statistically significant differences should be interpreted cautiously in the context of the limited sample size. In addition, the median follow-up duration was approximately one month, and although some patients had follow-up visits beyond this interval, longer-term follow-up data were not consistently available for all patients and therefore were not included in the current analysis. As a result, this study primarily reflects early postoperative outcomes and may underestimate clinically important long-term events, including hernia recurrence, chronic postoperative pain, and late complications. We did not assess chronic postoperative pain at follow-up because the dataset lacked consistent long-term outcome information. Whether robotic surgery offers potential benefits in reducing chronic pain remains uncertain and requires confirmation through longer-term follow-up studies. Furthermore, cost analyses and long-term outcomes such as chronic pain or quality of life were not assessed, restricting the evaluation to early postoperative results. Finally, surgeon selection bias and differences in learning curves for robotic and laparoscopic techniques may have influenced the observed outcomes.

## Conclusions

Robotic-assisted inguinal hernia repair was associated with similar observed short-term outcomes compared with conventional laparoscopic repair in terms of postoperative complications, hernia recurrence, reoperation, length of hospital stays, and analgesic use. However, given the small number of robotic cases and limited follow-up duration, these findings should be interpreted cautiously. Future large-scale prospective studies with long-term follow-up are warranted to further clarify its potential benefits in inguinal hernia surgery, particularly in technically demanding cases.

## Data Availability

The original contributions presented in the study are included in the article/Supplementary Material, further inquiries can be directed to the corresponding author/s.
